# Reconfiguration strategy for fully actuated translational cable-suspended parallel robots

**DOI:** 10.3389/frobt.2023.1112856

**Published:** 2023-02-06

**Authors:** Jason Bettega, Giovanni Boschetti, Giulio Piva, Dario Richiedei, Alberto Trevisani

**Affiliations:** ^1^ Department of Management and Engineering, University of Padova, Padua, Italy; ^2^ Department of Industrial Engineering, University of Padova, Padua, Italy

**Keywords:** cable-driven parallel robots, cable-suspended parallel robots, reconfigurability, reconfiguration strategy, wrench exertion capability

## Abstract

In Cable-Suspended Parallel Robots (CSPRs), reconfigurability, i.e., the possibility of modifying the position of the cable connection points on the base frame, is particularly interesting to investigate, since it paves the way for future industrial and service applications of CSPRs, where the base frame can also be replaced by mobile agents. This report focuses on fully actuated Translational Reconfigurable CSPRs (TR-CSPRs), i.e., reconfigurable CSPRs with a point mass end-effector driven by three cables. The objective of the work is twofold. First, it is shown that the Wrench Exertion Capability (WEC) performance index can be exploited to identify the configurations of the cable connection points optimizing a task-related performance in a single point or throughout the workspace, and hence to implement a workspace analysis. Then, by referring to the case of a TR-CSPR with a single reconfigurable connection point and in quasi-static working condition, an analytical approach is provided to reconfigure the robot while executing a task to avoid one of the paramount problems of cable robots: cable slackness. Brought together, the two contributions allow defining a reconfiguration strategy for TR-CSPRs. The strategy is presented by applying it to a numerical example of a TR-CSPR used for lifting and moving a load along a prescribed path: the use of the WEC allows analyzing the workspace and predicting if robot reconfigurability makes it possible to pass quasi-statically along all the points of a given path; then reconfigurability is exploited to avoid cable slackness along the path.

## 1 Introduction

### 1.1 State of the art

A Cable-Driven Parallel Robot (CDPR) is a parallel robot where the end-effector (EE) is actuated by cables whose lengths are set by winding them on motorized winches. Compared to classical parallel robots, CDPRs may present significant advantages, such as larger workspaces, higher dynamic performances, and higher payload capabilities. Moreover, cable winding systems are in general much cheaper to manufacture than the rigid links used as legs in parallel robots. These features make CDPRs interesting choices in industrial plants (Michelin et al., 2015), in telescoping operations (Nan et al., 2011), in urban mobility (Castelli et al., 2014), in home assistance (Merlet, 2008) and in human rehabilitation (Mao and Agrawal, 2011), just to mention a few representative field applications.

The number (*n*) and the layout of the cables reaching the EE of a CDPR considerably affect its performance. In order to fully or redundantly constrain the EE, it is required that *n* is at least greater by one than the number of the degrees of freedom ( $n_{DOF}$ ) of the EE: $n>n_{DOF}+1$ . Then, cables can balance an arbitrary external wrench with positive cable tensions and set a desired pose of the EE. Although a higher *n* improves CDPR performances in terms of workspace extension, payload capability and dynamic performance, the likelihood of cable collisions and the workspace obstruction are also increased. To overcome these issues Cable-Suspended Parallel Robots (CSPRs) may be adopted. Examples of CSPRs have been suggested for television shooting (Cone, 1985), payload lifting (Williams, 2005) and for construction systems (Bosscher et al., 2007). In such a topology, cables reach the EE only from above and the tension on cables is generated by gravity. In turn, CSPRs allow setting a desired pose of the EE even with $n=n_{DOF}$ . Apparently, such robots guarantee an unobstructed workspace under the EE but suffer from an underconstrained condition, which makes motion planning and control particularly challenging (Trevisani, 2013), especially when underactuation-related issues emerge (Idà et al., 2019).

Both in CDPRs and CSPRs, the possibility of modifying the configuration, meant as the position of the cable connection points on the base frame (also called exit-points), is particularly interesting, since it gives the possibility of significantly improving robot performances (Gagliardini et al., 2016a). The reconfiguration of the exit-points can be carried out in several ways, for instance by mobile agents (Jiang and Kumar, 2013; Masone et al., 2016; Six et al., 2017) and by mobile cranes (Nguyen et al., 2014) in Reconfigurable CSPRs (R-CSPRs), or through the reconfiguration of the whole base frame (Gagliardini et al., 2016b) in Reconfigurable CDPRs (R-CDPRs).

### 1.2 Aims of the paper

This work focuses the investigation on a spatial R-CSPR with a point mass EE ( $n_{DOF}=3$ ) actuated by three cables. Such a fully actuated CSPR is “translational” in the sense that the EE has only translational freedoms, which can be fully set by defining the cable lengths. The studied topology of Translational Reconfigurable CSPR (TR-CSPR) can be fairly simple to manufacture and could therefore find use in several service and industrial applications (e.g., as an industrial robotic crane or for cooperative transport with mobile agents); it also takes apparent and considerable advantage from reconfigurability which not only allows expanding the workspace but also achieving better task-related performance (e.g., payload lifting capacity) in specific positions within the workspace. Admittedly, these advantages can only be exploited if a reconfiguration strategy is developed.

The objective of this report, which introduces an opening study in this field, is twofold. First it is shown that the Wrench Exertion Capability (WEC) performance index proposed in (Boschetti and Trevisani, 2018) can be usefully exploited to identify the configuration of the exit-points which optimizes a task-related performance in a generic point of the robot workspace and, by iterating the investigation, to analyze the performance throughout the whole workspace. For sake of simplicity, but without loss of generality, the use of the WEC will be illustrated by considering a TR-CSPR with a single reconfigurable exit-point. Secondly, by assuming that the TR-CSPR operates in quasi-static conditions, the paper introduces an entirely analytical approach to reconfigure a single exit-point on-the-fly, while executing a task, avoiding one of the paramount problems of cable robots: cable slackness. It should be noted that the quasi-static working condition considered is quite popular in the case of R-CDPRs (see, for example (Gagliardini et al., 2016a)) and fits particularly well when CSPRs are used for handling heavy loads.

The analytical reconfiguration approach proposed, combined with the task-related workspace analysis based on the WEC, allow defining a reconfiguration strategy for TR-CSPRs which is then applied to an illustrative numerical example: a pick-and-place task imposing the reconfiguration of one exit-point. Such reconfiguration is assumed possible just along a given straight path (e.g., on a rail). First, the use of the WEC allows predicting if robot reconfigurability makes it possible to complete the task within the workspace; then reconfigurability is exploited to avoid cable slackness along the path.

This brief research report is structured as follows: the methodology section introduces the two combined contributions of the reconfiguration strategy: sub-Section 2.1 deals with WEC application to TR-CSPRs, sub-Section 2.2 deals with the analytical reconfiguration method. In Section 3 the achievements of sub-Section 2.1 and sub-Section 2.2 are combined to address the simulation of a representative pick-and-place task: the results are shown and discussed in the same Section, while concluding remarks are given in Section 4.

## 2 Methods

### 2.1 The wrench exertion capability index applied to TR-CSPRs

#### 2.1.1 General formulation

A major advantage of reconfigurability is the possibility of modifying the workspace shape and the robot performances within it. Not only obstacles or cable collisions can be avoided (Gagliardini et al., 2016a) but robot key performances, such as the maximum payload or energy absorption, can be optimized. Indeed, the performances of a CDPR can be evaluated through several global or local indices that have been proposed in the literature (see, for example (Gouttefarde et al., 2007; Duan and Duan, 2014) and the references therein). By reconfiguring the exit-points of a R-CDPR, not only global but also local properties can be altered considerably to meet some desired requirements.

The capability of a TR-CSPR of balancing an external action (e.g., payload or environmental forces) is a relevant example of performance that can be optimized by reconfiguration. The WEC index proposed in (Boschetti and Trevisani, 2018) provides an evaluation of the maximum external wrench that the EE of a CDPR can bear along a given direction of interest and it is first applied here to TR-CSPRs.

The WEC is a local performance index that computes the maximum wrench component the cables can exert on the EE along a prescribed direction while maintaining assigned wrench components on the remaining directions and taking explicitly into account the lower ( $\underline{t}$ , generally greater than zero) and upper ( $\bar{t}$ , usually depending on cable and motor physical properties) cable tension limits.

For translational CDPRs, just forces rather than combined forces and torques (wrenches) are considered applied to the EE. Then, the general formulation presented in (Boschetti and Trevisani, 2018) can be applied straightforwardly to TR-CSPRs actuated by *n* cables converging in the EE, however, reconfigurability makes the optimization problem non-linear: the computation of the WEC index imposes the solution of a non-linear optimization problem where cable tensions and the Cartesian coordinates of the movable exit-points are the unknowns. Let identify the spatial directions of the cable forces by the unit vectors $u_{i}$ , with i=1,…,n . Under the hypotheses of massless and inextensible cables, the *i-th* cable force is directed as the vector associated to the *i-th* cable, which connects the Cartesian position $p\in R^{3}$ of the EE to the Cartesian position $a_{i}\in R^{3}$ of the *i-th* exit-point; therefore, its direction is defined by $u_{i}=\frac{a_{i}-p}{\left\|a_{i}-p\right\|}$ . The matrix collecting vectors $u_{i}$ is referred to as the structure matrix S :

$$S\left(a\right)=\left[\begin{array}{cc} \begin{array}{cc} u_{1}\left(a_{1}\right) & u_{2}\left(a_{2}\right) \end{array} & \begin{array}{cc} \cdots & u_{n}\left(a_{n}\right) \end{array} \end{array}\right]\in R^{3\times n}$$ (1)

Where a is the set of Cartesian coordinates of the reconfigurable exit-points and, in the general case, $a=a_{1},\ldots ,a_{n}$ . For sake of clarity, in Eq. 1, and in all the equations below, the dependance of vectors, and matrices from the sole unknowns of the problem is explicitly shown.

Through $S\left(a\right)$ and by gathering the cable forces $t_{i}$ into vector t , the *n*-dimensional cable tension space is related to the three-dimensional space of the EE. If the equilibrium of the EE is considered, in addition to the force exerted by the cables ( $S\left(a\right)t$ ), the EE is subject to the global external force denoted by $w_{e}$ , which usually comprises the gravitational force $w_{g}$ and other external forces $w_{f}$ :

$$w_{e}=w_{g}+w_{f}$$ (2)

Let $\tilde{w}\left(a,t\right)$ denote the total force acting on the EE:

$$\tilde{w}\left(a,t\right)≜S\left(a\right)t+w_{e}$$ (3)

It can be recast as:

$$\tilde{w}\left(a,t\right)≜W\left(a\right)\tilde{t}$$ (4)

where the structure matrix $S\left(a\right)$ is augmented with vector $w_{e}$ yielding to the wrench matrix $W\left(a\right)=\left[\begin{array}{cc} S\left(a\right) & w_{e} \end{array}\right]$ , which multiplies the vector of the generalized cable forces $\tilde{t}={\left[\begin{array}{cc} t^{T} & 1 \end{array}\right]}^{T}$ . $W\left(a\right)$ can be also interpreted as the matrix that collects the three row vectors, namely $W\left(a\right)={\left[\begin{array}{ccc} {w\left(a\right)}_{x}^{T} & {w\left(a\right)}_{y}^{T} & {w\left(a\right)}_{z}^{T} \end{array}\right]}^{T}$ , that projects $\tilde{t}$ onto the axes of the global reference frame. Each row vector can be chosen to evaluate the force exertion capability of the TR-CSPR along a Cartesian direction. Following a similar reasoning, by means of a proper rotation matrix **R**, it is possible to evaluate the force exertion capability along any arbitrary direction **d**:

$$RW\left(a\right)={\left[\begin{array}{ccc} {w\left(a\right)}_{d}^{T} & {w\left(a\right)}_{o_{1}}^{T} & {w\left(a\right)}_{o_{2}}^{T} \end{array}\right]}^{T}$$ (5)

When the rotation matrix is employed, two additional orthogonal directions come along with **d**: $o_{1}$ and $o_{2}$ . These directions, taken together, define a new Cartesian reference frame on which the total wrench $\tilde{w}\left(a,t\right)$ is projected: $R\tilde{w}\left(a\right)={\left[\begin{array}{ccc} {\tilde{w}}_{d}\left(a,t\right) & {\tilde{w}}_{o_{1}}\left(a,t\right) & {\tilde{w}}_{o_{2}}\left(a,t\right) \end{array}\right]}^{T}$ . Hence, the computation of the WEC_**d**_ is carried out by solving the following optimization problem:

$${WEC}_{d}≜\max_{t,a}\left({\tilde{w}}_{d}\left(a,t\right)={w\left(a\right)}_{d}^{T}\tilde{t}\right)s.t.\left\{\begin{array}{c} \left[\begin{array}{c} {w\left(a\right)}_{o_{1}}^{T}\\ {w\left(a\right)}_{o_{2}}^{T} \end{array}\right]\tilde{t}=\left[\begin{array}{c} {\tilde{w}}_{o_{1}}\left(a,t\right)\\ {\tilde{w}}_{o_{2}}\left(a,t\right) \end{array}\right]={\tilde{w}}_{r}\\ a_{i}\in \Sigma_{i}i=1,\ldots ,n\\ \underset{–}{t}<t_{i}<\bar{t}i=1,\ldots ,n \end{array}\right.$$ (6)

which consists in finding **a** and **t** that maximize the force exerted along the direction of interest **d** ( ${w\left(a\right)}_{d}^{T}\tilde{t}$ ), while assigning given values ${\tilde{w}}_{r}$ (typically, but not necessarily, null values) to the forces along the directions $o_{1}$ ; $o_{2}$ , keeping the Cartesian coordinates of $a_{i}$ within the set of allowable configurations $\Sigma_{i}$ and keeping the cable tensions within $\underline{t}$ ; $\bar{t}$ . The optimization problem is non-linear because of reconfigurability which makes **W** a function of the unknowns **a**. Additionally, such non-linear terms also lead to a non-convex cost function and to non-convex constraints representing the force equilibria along the directions $o_{1}$ ; $o_{2}$ . In contrast, computing the WEC for fixed positions of the exit-points, as proposed in (Boschetti and Trevisani, 2018), leads to a linear and convex optimization problem whose solution is straightforward.

#### 2.1.2 Example of WEC computation for a representative TR-CSPR

**FIGURE 1:**
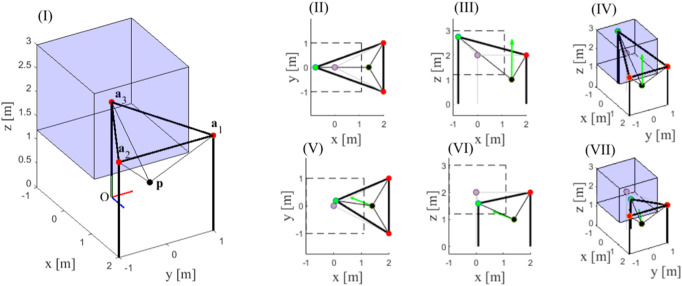
Nominal configuration of the TR-CSPR and optimal position of the reconfigurable exit-point for two different choices of the direction d of interest.

In this section, the general formulation of Eq. 6 is particularized for a three-cable TR-CSPR with a single reconfigurable exit-point. The TR-CSPR is shown in Figure 1I in a sample configuration, that is here discussed in detail: the EE, depicted as a black dot, has a mass $m_{ee}=3$ kg (assumed comprehensive of the payload) and it is placed at $p={\left[\begin{array}{ccc} 1.4 & 0 & 1 \end{array}\right]}^{T}$ m; the fixed exit-points are placed at $a_{1}={\left[\begin{array}{ccc} 2 & 1 & 2 \end{array}\right]}^{T}$ m; $a_{2}={\left[\begin{array}{ccc} 2 & -1 & 2 \end{array}\right]}^{T}$ m while the reconfigurable exit-point is initially placed at $a_{3}={\left[\begin{array}{ccc} 0 & 0 & 3 \end{array}\right]}^{T}$ m (denoted as the nominal position). The Cartesian coordinates of the reconfigurable exit-point $a_{3}={\left[\begin{array}{ccc} a_{3x} & a_{3y} & a_{3z} \end{array}\right]}^{T}$ can be independently changed within a rectangular cuboid $\Sigma_{3}$ defined through the respective allowable ranges [-1, 1.1] m [-1, 1] m and [1.2, 3] m. The components of $a_{3}$ are the unknowns of the problem together with the three cable tensions collected in **t**. The lower and upper cable tension limits are set to $\underline{t}$ = 0.1 N and $\bar{t}=100$ N. In Figure 1I all the exit-points are depicted in red, the reconfigurable one is in its nominal position and $\Sigma_{3}$ is shaded in blue. The global Cartesian reference frame Oxyz is shown too.

As a reasonable choice for this robot architecture, attention is paid to the maximum upward force that the cables can exert on the EE, the so-called WEC_**z**_, while keeping null force components along the horizontal directions ( ${\tilde{w}}_{r}={\left[\begin{array}{cc} 0 & 0 \end{array}\right]}^{T}$ N) and bounded cable tensions (with reference to the notation adopted in sub-Section 2.1.1, in this case; $d=z,o_{1}=x$ ; $o_{2}=y$ ). The following non-linear optimization problem is therefore stated:

$${WEC}_{z}≜\max_{t,a_{3}}\left({\tilde{w}}_{z}\left(a_{3},t\right)={w\left(a_{3}\right)}_{z}^{T}\tilde{t}\right)s.t.\left\{\begin{array}{c} \left[\begin{array}{c} {w\left(a_{3}\right)}_{x}^{T}\\ {w\left(a_{3}\right)}_{y}^{T} \end{array}\right]\tilde{t}=\left[\begin{array}{c} {\tilde{w}}_{x}\left(a_{3},t\right)\\ {\tilde{w}}_{y}\left(a_{3},t\right) \end{array}\right]={\tilde{w}}_{r}\\ a_{3}\in \Sigma_{3}\\ \underset{–}{t}<t_{i}<\bar{t}i=1,\ldots ,3 \end{array}\right.$$ (7)

The non-convex optimization problem formulated in Eq. 7 imposes the use of global optimization routines to get rid of the presence of several local minima and hence to find the actual optimal solution (Richiedei et al., 2019; Belotti et al., 2020). In this work, the standard *GlobalSearch* routine of Matlab is used to generate the set of initial guesses through an efficient and reliable scatter-search algorithm, while the gradient-based, *fmincon* interior-point solver is used as the local solver.

The analysis of such a sample position leads to ${WEC}_{z}=163.2$ N, by exploiting the optimal reconfiguration shown in Figure 1II, Figure 1III and Figure 1IV, where all the cable tensions reach the upper bound $t_{1}=100$ N, $t_{2}=100$ N, and $t_{3}=100$ N. The optimal position of the reconfigurable exit-point is painted in green, while its nominal position is painted in grey, the fixed exit-points are shown in red. Additionally, Figure 1II shows a top view of the TR-CSPR with the boundaries of $\Sigma_{3}$ sketched in dashed line, Figure 1III shows a side view, and finally, Figure 1IV reports a three-dimensional view, with $\Sigma_{3}$ shaded in blue. The green arrow applied to the EE represents $\tilde{w}\left(a_{3},t\right)$ , which has only an upward component.

Just to show how the optimal reconfiguration depends on the direction of interest, and hence on the task features, at the same position **p** of the EE the WEC is now computed considering another arbitrary direction $d={\left[\begin{array}{ccc} -0.88 & 0.34 & 0.32 \end{array}\right]}^{T}$ . The computation of WEC_**d**_ allows determining the optimal configuration assuring the exertion of the maximum force along direction **d** while maintaining null force components along $o_{1}$ and $o_{2}$ ( ${\tilde{w}}_{r}={\left[\begin{array}{cc} 0 & 0 \end{array}\right]}^{T}$ N). In this case such maximum force is equal to 90.3 N and the optimal configuration of the exit-point is depicted in Figure 1V; Figure 1VI and Figure 1VII with apparent meaning. Cable tensions take the following values $t_{1}=27$ N, $t_{2}=0.4$ N, and $t_{3}=100$ N.

**FIGURE 2:**
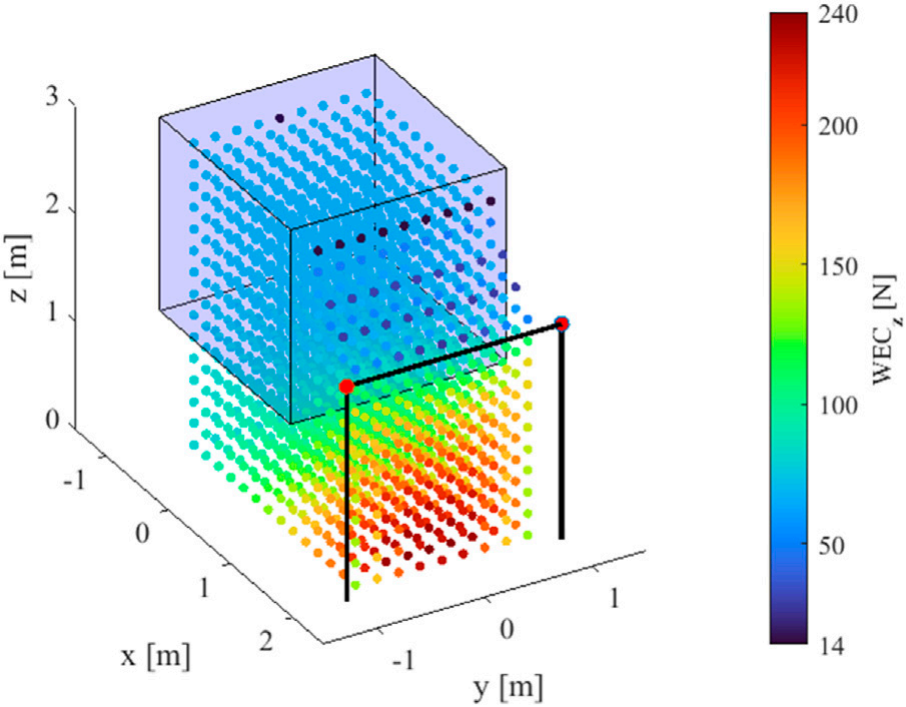
Results of a WEC_z_-based workspace analysis.

In general, by repeating the computation of the WEC_**d**_ at all the positions where equilibrium can be maintained by the EE, it is possible to perform a thorough task-related analysis of the performance achievable within the whole workspace. For example, the WEC_**z**_ analysis discussed above can be repeated in the Statically Feasible Workspace (SFW) of the TR-CSPR to analyze how the robot load-carrying capability changes as the EE changes position. The SFW is defined as the set of the EE poses for which static equilibrium against gravity can be obtained using a limited range of cable tensions (Trevisani, 2013). The results of such an analysis are shown in Figure 2: the three coordinates of the EE $p={\left[\begin{array}{ccc} p_{x} & p_{y} & p_{z} \end{array}\right]}^{T}$ change within the ranges [-1, 2] m [-1, 1] m and [0, 3] m with the step size 0.2 m in the **x**, **y**, and **z** directions, respectively. The investigated positions of the EE are depicted and painted in accordance with the values taken by the WEC_**z**_; for sake of clarity, $\Sigma_{3}$ is shown too. The predicted performance within the SFW can be immediately recognized by the color of the dots.

The result of this analysis can be exploited to preliminary assess whether a task can be carried out in a part of interest of the workspace. For example, the possibility of moving quasi-statically a load can be easily verified through WEC_**z**_: if the EE passes through positions where the WEC_**z**_ takes values greater than the load, there exists at least one configuration of the TR-CSPR which assures static equilibrium.

In the end, the WEC-based analysis appears useful to carry out preliminary investigations of task-related performances throughout the workspace, while it is not suitable to manage continuous exit-point reconfigurations for two reasons: firstly, the non-linear nature of the WEC formulation imposes a strong computational effort which is not compatible with real-time implementations; secondly, the optimal configurations computed in adjacent positions of the EE can differ significantly and could lead to discontinuous reconfigurations during the execution of a continuous displacement of the EE.

### 2.2 Reconfiguration method avoiding cable slackness

A method is therefore needed to reconfigure the exit-point continuously, while the EE is moving. In this section, by referring to the three-cable TR-CSPR with a single reconfigurable exit-point discussed above, and under the assumption that the TR-CSPR displaces a known load quasi-statically, an entirely analytical solution is proposed to move the exit-point continuously and to avoid cable slackness. This strategy still has some considerable limitations and lacks generality, but represents a first attempt to exploit continuous reconfigurability through an analytical and efficient strategy.

By assuming the static equilibrium of the EE $\tilde{w}={\left[\begin{array}{ccc} 0 & 0 & 0 \end{array}\right]}^{T}$ N and absence of external forces ( $w_{f}={\left[\begin{array}{ccc} 0 & 0 & 0 \end{array}\right]}^{T}$ N) excluding gravity, Eq. 3 can be rewritten as:

$$-St=w_{g}$$ (8)

where $w_{g}=-m_{ee}g$ and the gravity vector is defined as $g={\left[\begin{array}{ccc} 0 & 0 & g \end{array}\right]}^{T}$ , with g=9.81 m/s^2^. The cable tensions can be computed through:

$$t=-m_{ee}Vg$$ (9)

where $V=-S^{-1}$ . By writing $V=\left[\begin{array}{ccc} v_{1} & v_{2} & v_{3} \end{array}\right]$ , with $v_{1},v_{2},v_{3}\in R^{3}$ , Eq. 9 can be reformulated as:

$$t=-{gm}_{ee}v_{3}$$ (10)

Since *g* and $m_{ee}$ are known, Eq. 10 highlights that cable tensions are uniquely defined when $v_{3}$ is known. By denoting as $u_{ij}$ the arbitrary entry of **S**, whose columns are the unit vectors representing the cable directions, and where $u_{ij}$ is the *j-th* component of vector $u_{i}$ , then the expression of $v_{3}$ is achieved by the analytical development of $S^{-1}$ :

$$v_{3}=\frac{1}{\det\left(S\right)}\left[\begin{array}{c} u_{21}u_{32}-u_{31}u_{22}\\ u_{31}u_{12}-u_{11}u_{32}\\ u_{11}u_{22}-u_{21}u_{12} \end{array}\right]$$ (11)

with $\det\left(S\right)=u_{11}u_{22}u_{33}+u_{21}u_{32}u_{13}+u_{31}u_{12}u_{23}-u_{31}u_{22}u_{13}-u_{11}u_{32}u_{23}-u_{21}u_{12}u_{33}$ .

If the positions of the EE and of the exit-points are known, the entries of $v_{3}$ , and hence cable tensions, are unique. Such tensions, computed through Eq. 10, might take negative values for a given configuration. Considering $a_{3}$ the vector of the coordinates of the reconfigurable exit-point, the entries of **S** are not all known, since $u_{3}$ explicitly depends on $a_{3}$ , hence $\det\left(S\right)$ and $v_{3}$ are unknown. Then Eq. 10 can be rearranged to ensure positive cable tensions, yielding to the explicit definition of $a_{3}$ . It should be highlighted that a reconfigurable exit-point leads to operate with six unknowns ( $n_{un}=6$ ): the three entries of $u_{3}$ and the three entries of $a_{3}$ . Since three additional constraints ( $n_{constr}=n_{un}-n=3$ ) must be introduced to make the problem solvable, in this work a set of constraints is imposed when $t_{i}<\underline{t}$ :

1. Keeping $a_{3y}$ fixed
2. Keeping $a_{3z}$ fixed
3. Setting $t_{i}=\underline{t}$

Other choices may be possible and would be worth of investigation. In particular, the first and the second constraints can be readily changed to define other directions along which the exit-point can be displaced. However, through this choice, the problem solution is straightforward: $a_{3x}$ can be analytically determined through Eq. 10, ensuring positive cable tensions. Depending on which cable tension exceeds the lower bound $\underline{t}$ , different expressions are obtained: by defining

$$\begin{array}{cc} \Delta_{y}=p_{y}-a_{3y} & \Delta_{z}=p_{z}-a_{3z} \end{array}$$ (12)

o if $t_{1}<\underset{–}{t}$

$$a_{3x}=p_{x}-\frac{\Delta_{y}\left(-gm_{ee}u_{21}-\left(u_{21}u_{13}-u_{11}u_{23}\right)\underline{t}\right)-\Delta_{z}\left(u_{11}u_{22}-u_{21}u_{12}\right)\underset{–}{t}}{-gm_{ee}u_{22}+\left(u_{12}u_{23}-u_{22}u_{13}\right)\underset{–}{t}}$$ (13)

o if $t_{2}<\underset{–}{t}$

$$a_{3x}=p_{x}-\frac{\Delta_{y}\left(gm_{ee}u_{11}-\left(u_{21}u_{13}-u_{11}u_{23}\right)\underline{t}\right)-\Delta_{z}\left(u_{11}u_{22}-u_{21}u_{12}\right)\underset{–}{t}}{gm_{ee}u_{12}+\left(u_{12}u_{23}-u_{22}u_{13}\right)\underset{–}{t}}$$ (14)

o if $t_{3}<\underset{–}{t}$

$$\begin{array}{c} \begin{array}{l} \begin{array}{l} w_{1}=\left(u_{21}u_{13}-u_{11}u_{23}\right)\Delta_{y}\underset{–}{t}+\left(u_{11}u_{22}-u_{21}u_{12}\right)\Delta_{z}\underset{–}{t}\\ \alpha_{1}={\left(u_{12}u_{23}-u_{22}u_{13}\right)}^{2}{\underset{–}{t}}^{2}-{\left(-gm_{ee}\right)}^{2}{\left(u_{11}u_{22}-u_{21}u_{12}\right)}^{2} \end{array}\\ \begin{array}{l} \alpha_{2}=2w_{1}\left(u_{12}u_{23}-u_{22}u_{13}\right)\underset{–}{t}\\ \alpha_{3}=w_{1}^{2}-{\left(-gm_{ee}\right)}^{2}\left(\Delta_{y}^{2}+\Delta_{z}^{2}\right) \end{array} \end{array}\\ \begin{array}{l} \begin{array}{l} \beta_{1}=\alpha_{1}\\ \beta_{2}=-\alpha_{2}-2\alpha_{1}p_{x} \end{array}\\ \begin{array}{c} \beta_{3}=\alpha_{3}+\alpha_{2}p_{x}+\alpha_{1}p_{x}^{2}\\ \Delta =\beta_{2}^{2}-4\beta_{1}\beta_{3} \end{array} \end{array} \end{array}$$ (15)

and $a_{3x}=\frac{-\beta_{2}\pm \sqrt{∆}}{2\beta_{1}}$ .

By knowing $a_{3x}$ both $a_{3}$ and $u_{3}$ are defined, and therefore a new configuration with taut cables is achieved.

## 3 Results and discussion

The test case proposed in this Section involves the three-cable TR-CSPR with a single reconfigurable exit-point assumed performing heavy handlings tasks in quasi-static motion, like a robotic crane in an industrial plant. The workspace analysis presented in sub-Section 2.1.2 and the reconfiguration method in sub-Section 2.2 are here combined to prove that they provide a reconfiguration strategy which allows completing the task while preserving positive cable tensions.

**FIGURE 3:**
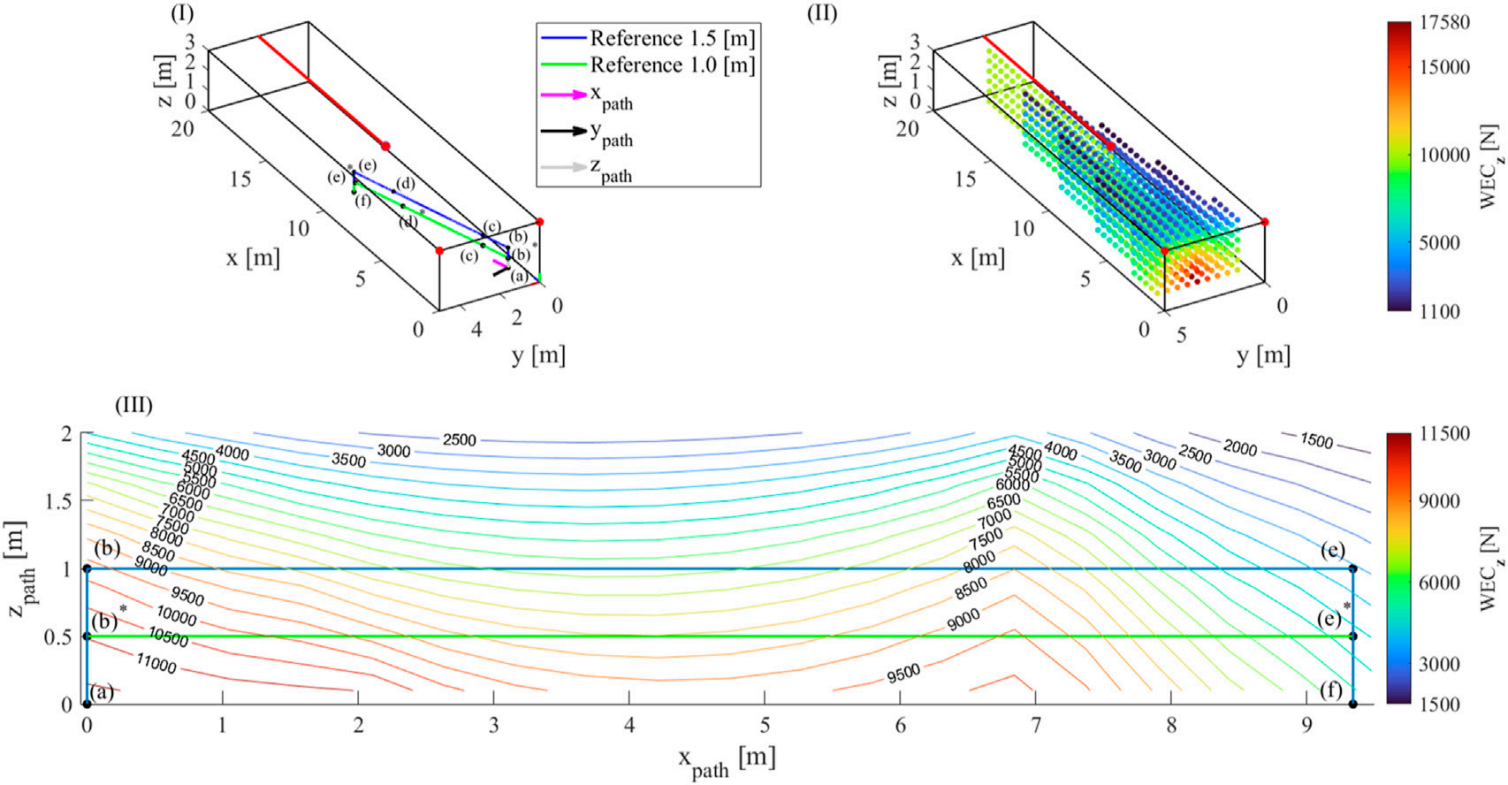
Representation of the considered paths (I), outcome of the WEC_z_ workspace analysis (II) and comparison among the desired paths and WEC_z_ values (III).

Figure 3 illustrates how the outcome of the WEC_**z**_ workspace analysis can be exploited to assess whether a desired path of the EE passes throughout feasible configurations of the robot. The advantage of such a workspace analysis is that it is valid regardless the mass that is intended to be lifted, whose change implies a modification of the external force in the **z** direction which can be straightforwardly compared to the WEC_**z**_ values computed along the path. Admittedly, if the task changed, and the resulting force one the EE was exerted along a different direction, the workspace analysis should be repeated. This should happen once at each task modification, but if the task does not change no WEC re-computation is needed.

The base frame of the robot has a length of 20 m (axis x), a depth of 5 m (axis y) and a height of 3 m (axis z) and is sketched in Figure 3I. The mass of the EE of the robot, including the payload, is $m_{ee}=450$ kg. The volume involved in the workspace analysis is bounded by the limits imposed on the Cartesian coordinates of the EE; $p_{x}$ varies in the range [1.5, 19.5] m; $p_{y}$ varies within [0.5, 4.5] m; $p_{z}$ varies within [0, 2.5] m and each direction is discretized with a step size of 0.5 m. All the exit-points are depicted in Figure 3I as red dots. The Cartesian coordinates of the fixed exit-points are $a_{1}={\left[\begin{array}{ccc} 0 & 0 & 3 \end{array}\right]}^{T}$ m; $a_{2}={\left[\begin{array}{ccc} 0 & 5 & 3 \end{array}\right]}^{T}$ m. The Cartesian coordinates of the reconfigurable exit-point are $a_{3}={\left[\begin{array}{ccc} a_{3x} & 2.5 & 3 \end{array}\right]}^{T}$ m, with $a_{3x}=9$ m in the initial position. Therefore, it is assumed that $\Sigma_{3}$ is a segment (painted in red in the figure): in particular, $a_{3x}$ can range between 9 m and 20 m. The cable tension limits are: $\underline{t}$ = 1 N and $\bar{t}=10^{4}$ N. The feasible workspace of the TR-CSPR, shown in Figure 3II, is identified by means of the computation of the WEC_**z**_ by changing **p** through its grid.

The results of Figure 3II are exploited to assess if a desired path passes through positions where the WEC_**z**_ is greater than the downwards force acting on the EE. As an example, two different paths are analysed. The desired task concerns the displacement of a payload from a pick location $\left(a\right)={\left[\begin{array}{ccc} 1 & 1 & 0.5 \end{array}\right]}^{T}$ m to a placement location $\left(f\right)={\left[\begin{array}{ccc} 10 & 3.5 & 0.5 \end{array}\right]}^{T}$ m: both locations are inside the feasible workspace.

In order to evaluate each task, auxiliary reference frames (x_path_y_path_z_path_) are introduced (see Figure 3I with origin in (a) and axis x_path_ oriented along the segment from (a) to (f). In the first considered path, the payload is lifted up to 1.5 m from the grasp location: a three-dimensional representation of the path and a planar one in the x_path_z_path_-plane are shown in blue respectively in Figure 3I and Figure 3III. In the second considered path, the payload is lifted up to 1 m, as shown in green in Figure 3I and Figure 3III. Figure 3III is enriched with WEC_**z**_ isolines drawn in the x_path_z_path_-plane proving that the blue path is unfeasible, since, for example, at the position e), the maximum WEC_**z**_ that can be exerted by the robot is less than the EE weight or downwards component of $w_{g}$ (3596 N vs. 4414.5 N). So, the preliminary WEC_**z**_ analysis shows that there is no reconfiguration which can prevent cables from exceeding tension limits along the path. Conversely, the green path is feasible since at no location the weight of the end effector exceeds the WEC_**z**_.

**FIGURE 4:**
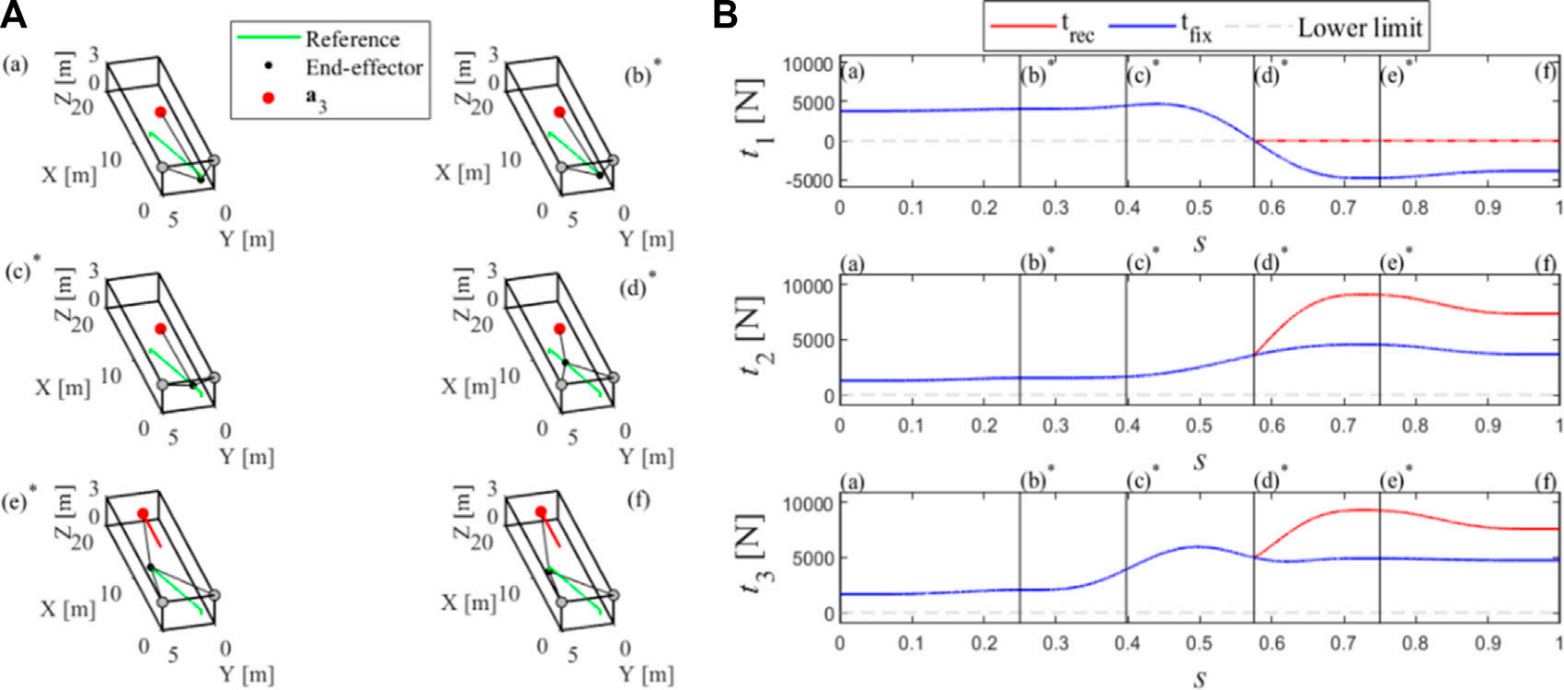
Reconfiguration of the exit-point **(A)** and cable tensions **(B)** along the path.

Let *s* be the curvilinear abscissa describing the displacement from (a) to (f) through all the *via* points. Figure 4 shows, on the left, the needed reconfiguration of the exit-point computed by the reconfiguration method in sub-Section 2.2, and, on the right, the cable tensions along the task, in both the cases that $a_{3x}$ is kept constant (t_fix_: blue line) or reconfigured (t_rec_: red line). Notice that if the exit-point was not reconfigured, cable tension $t_{1}$ would reach $\underline{t}$ at (d)^*^, which is therefore the point from which the reconfiguration method starts modifying the exit-point position, making the path accomplishment possible.

## 4 Conclusion and perspectives

This brief research report addresses two issues related to a fully actuated TR-CSPRs. First, it is shown that the WEC index can be successfully exploited to identify configurations of the exit-points optimizing task-related performances and to perform preliminary evaluations of the feasibility of given tasks within the workspace. Secondly, by focusing on a generic TR-CSPR actuated by three cables and with one reconfigurable exit-point which can be moved along a straight segment, it introduces an analytical reconfiguration method which can manage the displacement of the exit-point so as to assure positive tensions in all the cables during the whole task.

Clearly, an opening study is presented, but the use of the WEC for a preliminary evaluation of the workspace and of the feasibility of a task, combined with the implementation of an analytical approach capable of managing exit-point reconfiguration during task execution in a way which overtakes usual cable limitations, delineates an effective reconfiguration strategy for TR-CSPRs. The applicability of the strategy to more challenging R-CSPR topologies may not be trivial, in particular as far as the reconfiguration method is concerned, but certainly, worth investigating.

## Data Availability

The raw data supporting the conclusions of this article will be made available by the authors, without undue reservation.
